# Aberrant regulation favours matriptase proteolysis in neoplastic B-cells that co-express HAI-2

**DOI:** 10.1080/14756366.2019.1577831

**Published:** 2019-02-19

**Authors:** Yi-Lin Chiu, Yi-Ying Wu, Robert B. Barndt, Yee Hui Yeo, Yu-Wen Lin, Hou-Ping Sytwo, Huan-Cheng Liu, Yuan Xu, Bailing Jia, Jehng-Kang Wang, Michael D. Johnson, Chen-Yong Lin

**Affiliations:** a Lombardi Comprehensive Cancer Center, Department of Oncology, Georgetown University, Washington, DC, USA;; b Department of Biochemistry, National Defense Medical Center, Taipei, Taiwan;; c Division of Hematology/Oncology, Department of Internal Medicine, Tri-Service General Hospital, National Defense Medical Center, Taipei, Taiwan;; d School of Medicine, National Defense Medical Center, Taipei, Taiwan;; e Langley High School, McLean, VA, USA;; f Department of Gastroenterology, Henan Provincial People’s Hospital, Zhengzhou, China

**Keywords:** Matriptase, protease inhibition, HAI-1, HAI-2, B-cell lymphoma, pericellular proteolytic activity

## Abstract

Matriptase is ectopically expressed in neoplastic B-cells, in which matriptase activity is enhanced by negligible expression of its endogenous inhibitor, hepatocyte growth factor activator inhibitor (HAI)-1. HAI-1, however, is also involved in matriptase synthesis and intracellular trafficking. The lack of HAI-1 indicates that other related inhibitor, such as HAI-2, might be expressed. Here, we show that HAI-2 is commonly co-expressed in matriptase-expressing neoplastic B-cells. The level of active matriptase shed after induction of matriptase zymogen activation in 7 different neoplastic B-cells was next determined and characterised. Our data reveal that active matriptase can only be generated and shed by those cells able to activate matriptase and in a rough correlation with the levels of matriptase protein. While HAI-2 can potently inhibit matriptase, the levels of active matriptase are not proportionally suppressed in those cells with high HAI-2. Our survey suggests that matriptase proteolysis might aberrantly remain high in neoplastic B-cells regardless of the levels of HAI-2.

## Introduction

The type 2 transmembrane serine protease matriptase is expressed by approximately half of all non-Hodgkin B-cell lymphomas and by virtually all chronic lymphocytic leukaemia, but is not expressed by normal B-lymphocytes^1^^,^^2^. This is in marked contrast to the situation in carcinomas where both the tumour cells and the normal epithelial cells from which the tumours are derived express comparable levels of matriptase^3^. Ectopic expression of matriptase by B-cell cancers may contribute to the tumour phenotype through the activation of growth factors, such as hepatocyte growth factor (HGF) and macrophage stimulating factor 1 (MSP-1), or down-stream protease systems thought to play a role in tumour progression, such as urokinase-type plasminogen activator (uPA)^1^^,^^4–6^. The matriptase-mediated activation of the HGF-cMet pathway has been demonstrated to play an essential role in the initiation and progression of squamous cell carcinoma in a transgenic mouse model^7^^,^^8^. Matriptase substrates such as these are synthesised primarily by stromal cells and secreted as pro-forms, the activation of which requires proteolytic cleavage. Furthermore, the membrane receptors for these secreted factors are commonly expressed by cancer cells. Activation of these substrates by matriptase may occur in the pericellular milieu, sometimes involving the plasma membrane of the cancer cells^1^. The levels of extracellular matriptase proteolytic activity could, therefore, be highly related to its potential role in the activation of these important cancer-related substrates.

Matriptase is synthesised as a zymogen form, which possesses weak intrinsic activity like many other serine proteases. This matriptase zymogen intrinsic activity does not act on the same substrates as the mature activated enzyme, and it does not form stable complexes with protein protease inhibitors. This is because the substrate binding pocket has not assumed its mature (activated) conformation and so there are only low-affinity interactions between the protease zymogen and its substrates or protease inhibitors^9^^,^^10^. This intrinsic activity does, however, confer upon matriptase zymogen the ability to undergo autoactivation as the primary mechanism by which it acquires full enzymatic activity^11^, although the precise logistics of the activation mechanism, and more importantly how it is controlled, remain to be elucidated. Matriptase autoactivation can occur spontaneously and the rate of activation can be accelerated by various extracellular environmental factors such as a mildly acidic environment, reduced chloride concentrations, or a more oxidising redox environment^12–14^. Tumour microenvironments are commonly acidic and/or oxidative, under which conditions matriptase zymogen activation may be enhanced. Following matriptase zymogen activation, the newly formed active matriptase is either rapidly inhibited by the formation of high-affinity complexes with hepatocyte growth factor activator inhibitor (HAI)-1 or is shed from the cell surface^15^. The level of matriptase proteolytic activity in the pericellular environment is, therefore, determined by the dynamic balance between zymogen activation and the inhibition of the newly generated active matriptase.

Although HAI-1 was initially identified as a conventional matriptase inhibitor^16^^,^^17^, this Kunitz-type inhibitor has been shown to be required for the normal synthesis and intracellular trafficking of matriptase^11^^,^^18^. The importance of this somewhat unusual relationship is supported by the ubiquitous co-expression of matriptase with HAI-1 in epithelial and carcinoma cells^4^^,^^19^ and the fact that concomitant deletion of matriptase rescues the placental and epidermal defects, and chronic inflammation caused by the genetic deletion of HAI-1 in mice^20–23^. In light of this, it was a surprise that many matriptase-expressing neoplastic B-cells were found to lack significant expression of HAI-1^1^^,^^2^. This suggested that neoplastic B-cells must be equipped with some other protease inhibitor to substitute for HAI-1, not only for the control of matriptase activity, but also to replace the HAI-1 chaperone function facilitating matriptase synthesis and intracellular trafficking. HAI-2, another membrane-associated Kunitz-type serine protease inhibitor, highly related to HAI-1^24^, has also been shown to be able to inhibit matriptase activity and to support matriptase synthesis and intracellular trafficking^25–28^. In the current work, we set out to determine and characterise the role of HAI-2 in the regulation of matriptase extracellular proteolytic activity in neoplastic B-cells.

## Material and methods

### CCLE database and statistical analysis tools

The Cancer Cell Line Encyclopaedia (CCLE) database includes RNA-seq-based gene expression data for 945 cancer cell lines. The data are publicly available for download from the University of California, Santa Cruz Xena browser (xena.ucsc.edu/), where the raw expression data have been recomputed based on a standard pipeline to minimise differences from distinct sources, to facilitate the comparison of specific gene expression among different cell lines from various tissue types. All of the ratio and the value of concerned gene expression described in the current study were downloaded and calculated based on the RPKM value presented in the CCLE database in UCSC XENA. Data analysis and presentation were performed using Prism software (GraphPad Software, Inc., La Jolla, CA). The association of the gene expression with tissue type was determined using the Pearson chi-square test.

### Cell lines

Non-Hodgkin lymphoma cell lines, including the Mantle cell lymphoma line Jeko-1, the diffuse large B-cell lymphoma lines OCI-LY3 and OCI-LY10, and the Burkitt lymphoma lines Daudi, Namalwa, and Ramos were obtained from the Tissue Culture Shared Resource (TCSR) at the Lombardi Comprehensive Cancer Center, Georgetown University (Washington, D.C.). The identity of these lines was confirmed by short tandem repeat fingerprinting conducted by the TCSR which also confirmed that the lines were free of mycoplasma contamination. These haematological cells were cultured in RPMI-1640 medium (Lonza, Walkersville, MD), supplemented with 10% foetal bovine serum (FBS) at 37 °C in a humidified atmosphere with 5% CO_2_.

### Western blot and mAbs

The sample prepared for immunoblot analysis included cell lysates and the control, and pH 6 buffers that the cells were incubated with (conditioned buffer). The cells were lysed with 1% Triton in PBS or with radioimmunoprecipitation assay (RIPA) buffer. DTNB (5,5-dithio-bis-(2-nitrobenzoic acid) (1 mM) and/or protease inhibitor cocktail (Roche, Mannheim, Germany) were also included in the lysis buffer. The addition of DTNB in the lysis buffer is to prevent the cleavage of disulphide linkages^29^, which is important for the preservation of activated matriptase complexes with the HAIs, particularly for samples containing reducing species, such as reduced glutathione in the cell lysates. Protein samples were resolved by 7.5% SDS-polyacrylamide gel electrophoresis (SDS-PAGE) without the addition of a reducing agent and without boiling the samples to preserve activated matriptase complexes with HAIs, transferred to nitrocellulose membranes, and subsequently probed with mAbs, as indicated. Immunoreactive regions were visualised using horseradish peroxidase-labeled secondary antibodies and Western Lightning ECL pro reagent (PerkinElmer, Waltham, MA) exposed to x-ray film. When quantitative analyses were needed, signal intensity of western blot band was obtained by densitometry and processed by Image J (https://imagej.net/ImageJ). The primary antibodies used were the total matriptase mAb M24, the activated matriptase-specific mAb M69, the HAI-1 mAb M19, and the HAI-2 mAbs DC16 and XY9, the generation and characterisation of which is described in our previous studies^19^^,^^30^^,^^31^.

### Acid-induced matriptase activation

Matriptase zymogen activation can be robustly induced by transiently exposing cells to a pH 6.0 buffer at room temperature^12–14^. In order to better evaluate the levels of active matriptase shed by the cells, which is the focus of this study, a modified assay was used in which a fixed number of cells relative to the volume of acidic buffer was used (5 × 10^5^ cells per 10 ul of buffer), and the incubation temperature was controlled to 37 °C. The neoplastic B-cells were washed with PBS twice and then incubated either in PBS, as the non-activation control, or 75 mM phosphate buffer, pH 6.0 for 10 min at 37 °C. The conditioned buffer was then separated from the cells by centrifugation using a Microfuge 22 R with Rotor F241.5P (Beckman Coulter) at 2000 rpm for 5 min at 4 °C in order to prevent rupture of the cells. The cell pellets were then lysed with the same volume of lysis buffer as the conditioned buffer collected, as described above.

### Immunodepletion

Conditioned buffer (200 µl) collected from Ramos cells after the induction of matriptase zymogen activation was incubated with the activated matriptase mAb M69 linked to Sepharose beads (15 µl drained beads) and mixed end-over-end in a cold room for 2 h. The supernatant was collected by centrifugation and referred to as the activated matriptase-depleted fraction.

### Matriptase amidolytic assay

For fluorogenic peptide substrate cleavage assays, samples of the shed fraction were incubated with (N-t-Boc)-Gln-Ala-Arg-AMC at a final concentration of 10 µM in a reaction buffer of 100 μl containing 20 mM Tris-HCl, pH 8.5. The increase in fluorescence resulting from the hydrolysis of the peptide substrate was recorded using a Wallac 1420 Victor 2 (Perkin Elmer^®^) microplate reader, using an excitation wavelength of 355 nm and measuring emission at 460 nm. The fluorescence signal was recorded every three minutes for 30–40 min.

### Gelatine zymography

For gelatine zymography, 1 mg/mL gelatine was co-polymerised in 7.5% SDS polyacrylamide gels. Details of the gelatine zymography assay have been described in our previous study^32^. Samples of the shed fraction were mixed with 5× sodium dodecyl sulphate (SDS) sample buffer containing no reducing reagent and incubated at room temperature for 5 min. The proteins were resolved on SDS-PAGE on polyacrylamide gels containing 1 mg/ml gelatine. The gelatine gels were washed with PBS containing 2.5% Triton X-100 to remove SDS and then incubated in pH 8.5 Tris buffer at 37 °C, overnight. The gels were stained with Coomassie Brilliant Blue R250 at room temperature for 1 h and then destained using 10% isopropanol and 10% acetic acid. Gelatine zymography is a standard method to detect enzymatic activity of some proteases and has been widely used in the field of matrix metalloproteases (MMPs)^33^. Interestingly, the SDS present in the loading buffer and gels, rather than denaturing the enzyme, is, in fact, able to induce the activation of pro-MMPs through a proposed mechanism called “cysteine switch”^34^. Matriptase contains 20 pairs of disulphide linkages and so is not denatured by 1% SDS under non-reducing and non-boiled conditions. Once the SDS is washed away by nonionic detergents, the matriptase proteolytic activity remains intact and can digest the gelatine in the gel. The potent matriptase gelatinolytic activity from either free active matriptase, or even the enzyme in complex with the reversible protease inhibitor HAI-1, has frequently been used to identify and characterise this membrane-bound serine protease^1^^,^^16^^,^^30^^,^^32^^,^^35–37^.

## Results

### Matriptase is co-expressed with HAI-2 more frequently than HAI-1 in human neoplastic B-cells in contrast to epithelial/carcinoma cells where matriptase is typically co-expressed with both HAI-1and HAI-2

**Figure 1.**
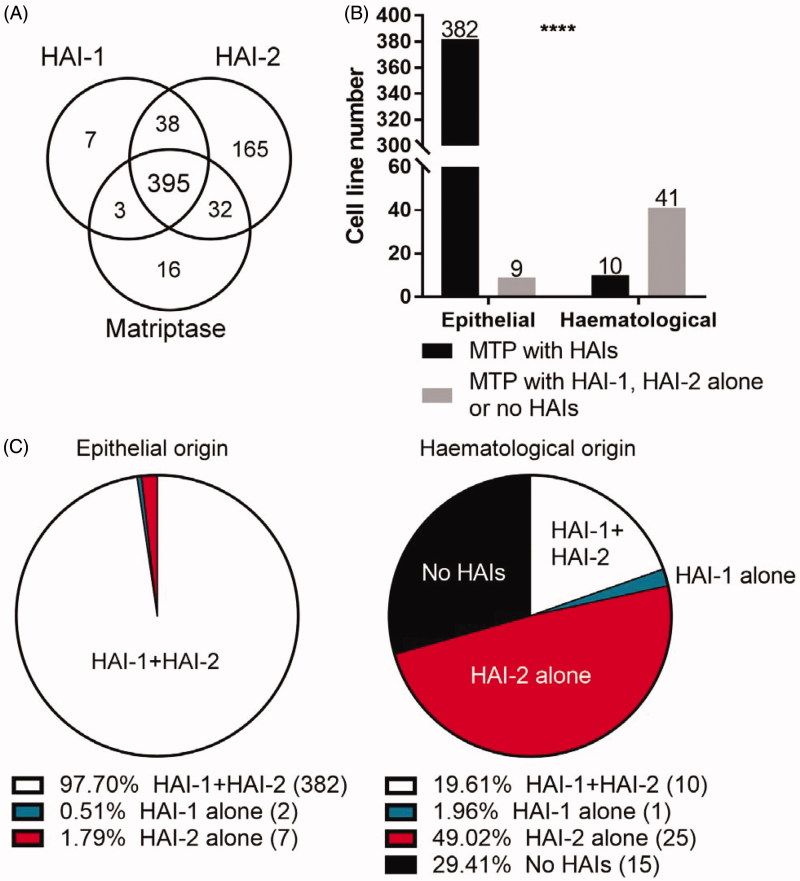
Matriptase could differentially be regulated between haematological and epithelial/carcinoma cells. (A) The mRNA expression levels for matriptase, HAI-1, and HAI-2 in a variety of cell lines were obtained from the CCLE databases. An RNA-seq reading count lower than 10 RPKM (Reads Per Kilobase Million) was identified as absent transcript expression. The number of cell lines expressing the three proteins and the number of the cell lines with overlapping expression of the three proteins are presented with their distribution within the circles. (B) The cell lines collected in the CCLE database were grouped into epithelial (carcinoma) and haematological (lymphoid and haematopoietic neoplasm) based on their cell-type origin. Chi-square testing was applied to compare differences between the ratio of matriptase with HAI-1, HAI-2 alone or no HAIs in epithelial origin and haematological origin. ****p < .001. (C) The ratio of matriptase to HAIs, HAI-1 alone, HAI-2 alone, or lack of HAIs co-expression was presented in the Pie chart of the epithelial origin or haematological origin, respectively.

In order to define the role played by HAI-2 in matriptase regulation in neoplastic B-cells, the global expression status of matriptase in relation to HAI-1 and HAI-2 at the mRNA level in haematological cancer cells versus epithelial/carcinoma cells was analysed in the 945 human cancer cell lines collected in the Cancer Cell Line Encyclopaedia (CCLE) database. This database is intended to capture the genomic diversity of human cancers and to be used as a robust preclinical model system for anticancer drug sensitivity prediction. As shown in Figure 1(A), matriptase mRNA was detected in 446 (47%) out the 945 lines collected in the database. Among the 446 matriptase-expressing lines, 391 (88%) are epithelial/carcinoma and 51 (12%) are haematological lines. For those epithelial/carcinoma lines expressing matriptase, 382 out of the 391 lines (98%) express both HAI-1 and HAI-2 together (Figure 1(B)), consistent with our previous study in which all 21 matriptase-expressing epithelial/carcinoma lines express both HAI-1 and HAI-2 at protein levels^19^. Interestingly, these data suggest that matriptase could also be under the tight control of the two Kunitz-type serine protease inhibitors in haematological cancer cells, as the vast majority of which tumours that express matriptase, it is co-expressed with both or either of HAIs (36 out of 51, 71%) (Figure 1(C)). In contrast to epithelial cancer lines, the co-expression of both HAI-1 and HAI-2 together was seen only in approximate one-fifth of these matriptase-expressing haematological cancer lines (10 out of 51; 20%) (Figure 1(B)). Instead, 25 out of the 51 haematological cancer lines (49%) express HAI-2 in the absence of HAI-1; only 1 out of the 51 lines (2%) express HAI-1 in the absence of HAI-2 (Figure 1(C)). The expression profile suggests while matriptase is co-expressed with the two Kunitz-type serine protease inhibitors, either both or alone, HAI-2 appears to be co-expressed with matriptase at higher frequency than HAI-1 in haematological cancer lines.

**Figure 2.**
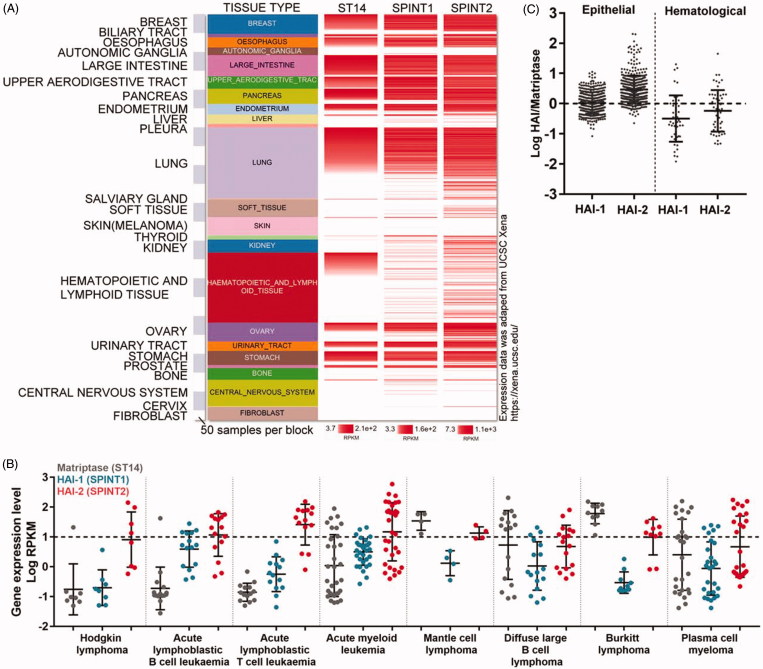
Imbalanced expression of matriptase relative to HAIs is worsening in haematological cancer cells versus carcinoma cells. (A) The relative mRNA expression levels of matriptase (st14), HAI-1 (spint1), and HAI-2 (spint2) in the whole cancer cell lines from CCLE were comprehensively retrieved, presented, and compared using the UCSC Xena Platform^41^. The amount of matriptase mRNA expression in each cell line was obtained and ranked from high to low. The mRNA expression levels of HAI-1 and HAI-2 were arranged in the order of the amount of matriptase expression. The size of each block represents 50 cell lines. The RNA-seq reading count is proportional to the depth of the red, as shown below each lane. (B) Matriptase, HAI-1, and HAI-2 mRNA expression levels (Log RPKM) of eight major blood cancer subclasses were retrieved from the histological type of “haematopoietic and lymphoid tissue classification” in the CCLE database. Horizontal dashed lines are used to distinguish the threshold for mRNA expression levels identified as proteins that may be expressed in cell lines. (C) The logarithm of HAI-1:matriptase or HAI-2:matriptase expression was calculated from the epithelial origin and haematological origin retrieved from CCLE database, respectively. The horizontal dotted line represents the ratio of HAIs:matriptase was 1:1.

The contrast in expression profile of matriptase in relation to HAIs in haematological versus epithelial/carcinoma cells can be further illustrated by the expression trend based on individual cells which were grouped by the organ origin with matriptase expression levels being presented in a descending manner (Figure 2(A)). In almost all epithelial/carcinoma cells regardless their organ origins, matriptase is closely expressed with high levels of both HAI-1 and HAI-2 together. Interestingly, for some matriptase-negative cells in which HAI-1 and HAI-2 are also expressed, the levels of HAI-1 tends to be lower than that in matriptase-expressing cells. This effect is not as noticeable for HAI-2 as it is for HAI-1. For example, high levels of HAI-2 but not HAI-1 is expressed in some matriptase-negative cells originating from the lung and soft tissues. It remains unclear what the role of the HAIs might be in these cells, and this is beyond the scope of the current study. Low HAI-1 expression was not only seen in matriptase-negative cells but also seen in matriptase-positive haematological cells. Although HAI-2 appears to be expressed at lower levels in matriptase-positive haematological cells compared to epithelial cells, the effect is not as marked as it is for HAI-1 (Figure 2(A)).

Haematological cancer cells can be further divided into several subtypes (Figure 2(B)). Matriptase is expressed at low levels in Hodgkin lymphoma cells, acute lymphoblastic B-cell leukaemia, and lymphoblastic T-cells leukaemia cells. Among those subtypes of haematological cancers with significant matriptase expression, neoplastic B-cells from Burkitt lymphoma tend to express HAI-2 in the absence of HAI-1 (Figure 2(B)). Taken together, the co-expression status of matriptase with respect to the HAIs in haematological cancer cells is somehow different from that found in epithelial cells with (1) reduced levels of HAI expression overall, and (2) increased frequency of higher levels of HAI-2 than HAI-1.

The enzymatic activity and pathophysiological function of a given protease can significantly depend on the ratio of the protease expression relative to that of its endogenous protease inhibitors. Thus, the HAI-1:matriptase mRNA ratio and the HAI-2:matriptase mRNA ratio were calculated for these matriptase-expressing cells and are presented on a logarithmic scale sorted by their cellular origins in a scatter plot (Figure 2(C)). In epithelial/carcinoma cells, matriptase tends to be expressed with similar levels of HAI-1 based on the relatively narrow distribution of the log HAI-1/matriptase ratio of between 0.5 and −0.5 among the majority of these epithelial/carcinoma cells, indicating that the HAI-1:matriptase mRNA ratio varies between 3 and one third. Epithelial/carcinoma cells tend to express HAI-2 at higher levels in relation to matriptase based on the distribution of the log HAI-2/matriptase ratio of between 0 and 1 among majority of these epithelial/carcinoma cells and between 1 and 2 among for some other lines, indicating that the HAI-2:matriptase mRNA ratio varies between 1 and 100. In contrast to the situation in epithelial/carcinoma cells, the distribution of log HAI/matriptase mRNA ratios was over a much larger range and shifted down below 0 for a significant proportion of cell lines of haematological origin (Figure 2(C)). These expression distribution patterns suggest that an imbalanced expression of matriptase favouring proteolysis might be present in haematological cancer cells. Furthermore, the higher ratio of log HAI-2/matriptase mRNA compared to log HAI-1/matriptase mRNA suggests that haematological cancer cells have a tendency to express higher levels of HAI-2 than HAI-1. The HAI-2 expression could be particularly important for those cells in which the log HAI-1/matriptase mRNA is very low and HAI-1 expression is, therefore, negligible. It is worth noting that the six lines with very highest HAI-1:matriptase ratio, in fact, resulted from very low matriptase expression rather than high HAI-1 expression.

### HAI-2 protein expression levels vary significantly in matriptase expressing neoplastic B-cells

**Figure 3.**
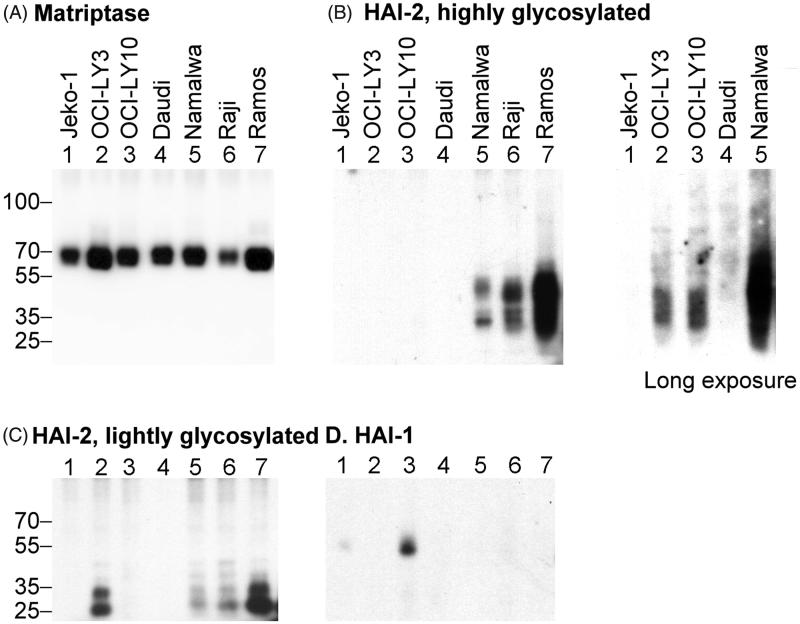
The co-expression status of HAI-1 and HAI-2 at protein levels in seven matriptase-expressing neoplastic B-cells. One million (106) cells from seven different matriptase-expressing neoplastic B-cell lines, as indicated, were analysed by immunoblot for protein levels of matriptase (A), the two species of HAI-2 (B and C), and HAI-1 (D). The expression of the highly glycosylated HAI-2 was also assessed with longer X-ray film exposure, as indicated.

In order to further characterise how matriptase proteolytic activity is regulated in haematological cancer cells, the levels of HAI-1 and HAI-2 protein expression in a panel of 7 different matriptase-expressing neoplastic B-cell lines were evaluated. The 7 lines express matriptase at relatively comparable levels with Ramos cells having the highest expression and Raji cells the lowest when lysates prepared from equal numbers of cells (10^6^) were assayed by western blot analysis (Figure 3(A), Matriptase). In epithelial/carcinoma cells, HAI-2 is expressed as one of two distinct species at a relatively constant ratio by different cell lines^31^. A species with extensive N-glycan branching can be detected using the mAb DC16 as a diffuse band of apparent molecular mass of 30–40 kDa (Figure 3(B), HAI-2, highly glycosylated); whereas the second species with oligomannose-type N-glycan can be detected by the mAb XY9 as a doublet with an apparent mass of ∼25-kDa (Figure 3(C), HAI-2, lightly glycosylated). Both HAI-2 species were detected in Namalwa, Raji, and Ramos cells with the highest level in Ramos and lowest levels in Namalwa cells, although the ratio between the two species was relatively consistent across the various lines (Figure 3(B,C), lanes 5, 6 and 7). OCI-LY 3 was an exception to this as although the HAI-2 species with oligomannose-type N-glycan was clearly detected (Figure 3(C), lane 2), the levels of the form with extensive N-glycan were much lower (Figure 3(B), lane 2). Very low-level expression of the HAI-2 species with extensive N-glycan could only be detected in OCI-LY3 and also OCI-LY10 cells after longer exposure of the western blot analysis (Figure 3(B), Long exposure). HAI-2 expression in Daudi cells was even lower than OCI-LY3 and OCI-LY10, and required the use of more protein and a longer exposure to be detected (Data not shown). No signal for HAI-2 was detected with Jeko-1 cells even after longer exposure in western blot analysis (data not shown). HAI-1 was only detected in Jeko-1 and OCI-LY10 at relatively low levels (Figure 3(D), HAI-1). No HAI-1 signal was observed for the remaining 5 lines even after longer exposure in the western blot analysis (not shown). The lack of HAI-1 protein expression in Daudi, Namalwa, Raji, and Ramos is consistent with their Burkitt lymphoma origin (Figure 2(B)). This analysis suggests that HAI-2 is more frequently expressed than HAI-1 in matriptase-expressing neoplastic B-cells and that the levels of HAI-2 relative to matriptase appear to vary significantly between different cell lines.

### HAI-2 can function as matriptase enzymatic inhibitor in neoplastic B-cells

The expression analysis at both the mRNA and protein levels described above indicates that HAI-2 might play a major role in the regulation of matriptase enzymatic activity in haematological cancer cells, particularly for those cancer cells in which HAI-1 expression is negligible^1^. While it has been shown that in solution, HAI-2 can inhibit active matriptase through the formation of a stable complex at comparable potency to that of HAI-1, the ability of HAI-2 to actually function as a matriptase inhibitor is, however, cell-type selective. For example, while HAI-2 and HAI-1 are expressed at high levels in 184 A1N4 human mammary epithelial cells and HaCaT human keratinocytes^19^, HAI-1, and not HAI-2, represents the predominant protease inhibitor for the control of matriptase proteolytic activity. In contrast, in breast cancer cells, matriptase enzymatic activity is under the control of both HAI-1 and HAI-2. This conclusion was drawn based on which HAIs were able to form stable protease-protease inhibitor complex with the nascent active matriptase after the induction of matriptase zymogen activation.

**Figure 4.**
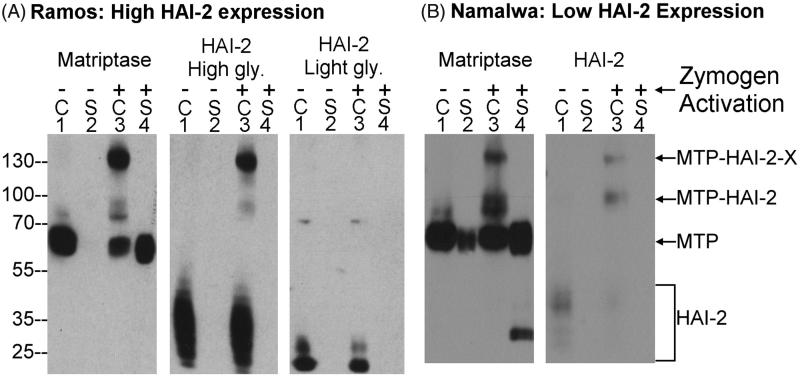
HAI-2 can function as matriptase enzymatic inhibitor in neoplastic B-cells. One million Ramos (A) and Namalwa cells (B) were transiently exposed to PBS as non-activation control (lanes 1 and 2) or a pH 6.0 buffer to induce matriptase zymogen activation (lanes 3 and 4). The cell lysates (lanes 1 and 3, indicated by C) and the conditioned buffer (lanes 2 and 4, indicated by S) were prepared to give identical final volumes. Equal volumes of these samples were analysed by western blot for matriptase and the highly glycosylated (High gly.) and lightly glycosylated (Light gly.) HAI-2 species. MTP-HAI-2-X stands for matriptase-HAI-2-protease X complexes, MTP-HAI-2 for matriptase-HAI-2 complex, MTP for matriptase. The immunoblot data presented are representative examples of at least three independent experiments.

In order to determine if HAI-2 can function as a matriptase enzymatic inhibitor at the cellular level in neoplastic B-cells, we took advantage of the well-characterised methods for the induction of matriptase zymogen activation and the rapid formation of stable complexes between the nascent active matriptase and the HAIs we have used previously. Matriptase zymogen activation was induced (or accelerated) by transient (20 min) exposure of these cells to a pH 6.0 buffer (Figure 4). The formation of stable HAI-2 complexes with the active matriptase generated by this exposure was used as a readout of the role of HAI-2 in matriptase inhibition. We began with Ramos cells which express high levels of HAI-2 and no detectable HAI-1 (Figure 4). Prior to the induction of matriptase zymogen activation, matriptase was detected as the 70-kDa zymogen form (Figure 4(A), Matriptase, lane 1). The two HAI-2 species were detected as monomers (Figure 4(A), HAI-2, lanes 1). Upon transiently exposing the cells to a pH 6.0 buffer, a significant proportion of matriptase zymogen was converted into two activated matriptase-HAI-2 complexes with apparent masses of ∼130- and 100-kDa, both of which were detected by the anti-matriptase mAb and the anti-HAI-2 mAb DC16 (Figure 4(A), middle panel, lane 3), but not by the anti-HAI-2 mAb XY9 (Figure 4(A), right panel, lane 3). Based on the size of the constituent molecules, the 100-kDa represents a matriptase-HAI-2 complex with 1:1 stoichiometry, whereas the 130-kDa complex is likely formed by one molecule of HAI-2 bound to matriptase and another as yet unidentified serine protease; a hypothesis supported by the fact that HAI-2 contains two functional Kunitz inhibitor domains. Regardless of the identity of this protease, the matriptase species in both the 130- and 100-kDa HAI-2 complexes are in the activated form. In addition to the western blot analysis presented here, the composition of these two matriptase-HAI-2 complexes has previously been verified by a combination of immunodepletion and western blot analysis^19^. These data suggest that the HAI-2 species with extensive N-glycan branching can function as a matriptase inhibitor in Ramos cells. Induction of matriptase zymogen activation also resulted in the formation of HAI-2 complexes in Namalwa cells, in which the level of HAI-2 and the ratio of HAI-2 relative to matriptase is noticeably lower than that in Ramos cells (Figure 4(B), comparing lanes 3 with lanes 1). These data suggest that the mature HAI-2 with extensive N-glycan branching but not the HAI-2 species with oligomannose-type N-glycan can function as a matriptase inhibitor in neoplastic B-cells. It is worth pointing out that the inability of the HAI-2 species with shorter N-glycan glycosylation to inhibit matriptase in a cell-based system, provides additional evidence that the functional relationship between matriptase and HAI-2 goes beyond the simple enzymatic relationship that can be observed in solution-based studies and their co-expression in the cells.

### Neoplastic B-cells shed enzymatically active matriptase into the extracellular milieu largely in proportion to the matriptase protein expression levels

The pathophysiological function of matriptase is likely dependent on its enzymatic activity which is capable of activating and processing downstream substrates. Previous studies have demonstrated that enzymatically active matriptase along with matriptase zymogen are rapidly shed from the surface of cells following the induction of zymogen activation^1^^,^^15^. As might be expected, the shedding of matriptase increases significantly with the induction of matriptase zymogen activation by pH 6.0 buffer exposure (Figure 4(A,B), Matriptase, comparing lanes 4 with lanes 2). In contrast, shedding of HAI-2 did not occur along with the induction of matriptase zymogen activation (Figure 4(A,B), HAI-2, comparing lanes 4 with lanes 2). In epithelial/carcinoma cells, HAI-2 is primarily localised inside the cells^19^^,^^31^. The lack of HAI-2 shedding might be consistent with an intracellular localisation, if neoplastic B-cells retain the subcellular localisation of HAI-2 observed in epithelial cells.

**Figure 5.**
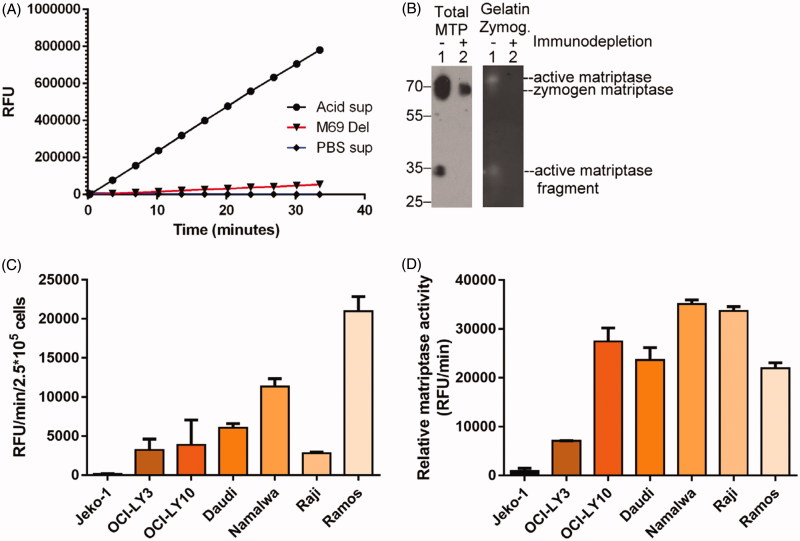
The levels of shed active matriptase are roughly correlated with the levels of matriptase expression. The conditioned buffer (shed fraction) was collected from Ramos cells following acid-induced matriptase zymogen activation. The shed fraction was then subjected to immunodepletion using the activated matriptase mAb M69 bound to Sepharose beads. The shed fraction (A: Acid sup; B: lanes 1), the activated matriptase-depleted shed fraction (A: M69 Del; B: lanes 2), and the control shed fraction (A: PBS sup) were analysed for tryptic activity using a synthetic fluorogenic substrate (A), for matriptase species by immunoblot using the total matriptase mAb (B: left panel, Total MTP), and gelatinolytic activity by gelatine zymography (B: right panel, Gelatine Zymog.). Half a million cells of each of the seven-different neoplastic B-cell lines were transiently exposed to a pH 6.0 buffer. The shed fraction was collected and analysed for the tryptic activity. The rate (RFU/min) of the tryptic activity is presented per 2.5 × 105 cells (C) or normalised to the level of matriptase expressed by these seven lines (D). RFU stands for relative fluorescent unit. The immunodepletion studies and matriptase enzymatic activity assays were conducted at least two times, and representative data are presented.

The levels of shed active matriptase in the conditioned buffer can be determined by assaying amidolytic activity through the cleavage of a synthetic fluorogenic peptide substrate (Boc-Gln-Ala-Arg-AMC). Activity was only detected in the conditioned buffer of cells exposed to a pH 6.0 buffer (Figure 5(A), Acid sup) and not the non-activation control PBS conditioned buffer (Figure 5(A), PBS sup). Confirmation that the proteolytic activity detected in the shed fraction is due to matriptase was provided by demonstrating that removal of the activated matriptase from the buffer by immune-depletion using activated matriptase-specific mAb M69-Sepharose beads, also depletes the amidolytic activity in the buffer (Figure 5(A), M69 Del). The matriptase species present in the shed fraction before and after the immune-depletion of active matriptase was further examined by immunoblot using the total matriptase mAb M24 (Figure 5(B), Total MTP, left panel) and by gelatine zymography (Figure 5(B), Gelatine Zymog., right panel). Matriptase species detected using the total matriptase mAb M24 include 70-kDa mature matriptase, and a 35-kDa fragment (Figure 5(B), left panel, lane 1), and gelatinolytic activity was also be detected at approximately the same location by gelatine zymography (Figure 5(B), right panel, lane 1). Immunodepletion of active matriptase species using the activated matriptase-specific mAb M69 removed both the majority of the 70-kDa and all the 35-kDa protein bands (Figure 5(B), left panel, lane 2) and the 70- and the 35-kDa gelatinolytic activities (Figure 5(B), right panel, lane 2). These data confirm the shedding of enzymatically active matriptase following induction of matriptase zymogen activation. In addition to active matriptase, the conditioned buffer also contained matriptase zymogen, which was not immunodepleted by the activated matriptase-specific mAb M69 (Figure 5(B), left panel, lane 2). It should be noted that the samples for immunoblot analysis and gelatine zymography were not treated with a reducing agent or boiled before loading on their respective gels (non-reducing and non-boiled conditions). Under these conditions, the light and heavy chains of activated matriptase remain associated by a disulphide linkage and both the active and zymogen forms of matriptase are, therefore, expected to be of a similar size.

In order to investigate how matriptase proteolytic activity is regulated in neoplastic B-cells, we next determined the levels of matriptase proteolytic activity shed from equal numbers (2.5 × 10^5^) of the seven neoplastic B-cell lines following induction of matriptase zymogen activation. As shown in Figure 5(C), the matriptase proteolytic activity shed by the various lines varied significantly, with Jeko-1 cells shedding a negligible amount, to a modest level from Raji cells and the highest level shed from Ramos cells. The lack of matriptase activity shed from Jeko-1 cells (Figure 5(C)) and the lack of detectable matriptase-HAI-1 complex (data not shown) suggest that Jeko-1 cells might not be able to activate matriptase in response to what is the most potent stimuli for matriptase zymogen activation in other systems^13^. As these different lines express different levels of matriptase (Figure 3(A)), we evaluated the relationship between matriptase expression and the level of shed matriptase proteolytic activity (Figure 5(D)). The ratio of total matriptase expression to matriptase proteolytic activity shed should provide some insight as to the important determinants of the release of proteolytic activity into the peri-cellular environment: IE – does more matriptase expression lead to more extracellular activity, is the level of HAI-2 and its ability to inhibit matriptase activity more important, or is there some factor that impacts the amount of total matriptase that can be activated? Interestingly, with the exception of Jeko-1 and OCI-LY3, the ratio of shed matriptase proteolytic activity to the total level of matriptase protein expressed was surprisingly similar for the remaining 5 neoplastic B-cell lines (Figure 5(D)). This was particularly surprising given the large differences in the levels of HAI-2 expressed by these lines: from very high levels in Ramos cells to very low levels in Daudi and OCI-LY10 cells (Figure 3(C)). This is at odds with the conventional belief that more HAI-2 expression should result in less matriptase proteolytic activity.

### In spite of its potent inhibitory activity against matriptase, an elevated HAI-2:matriptase ratio does not result in the effective suppression of extracellular matriptase proteolytic activity

**Figure 6.**
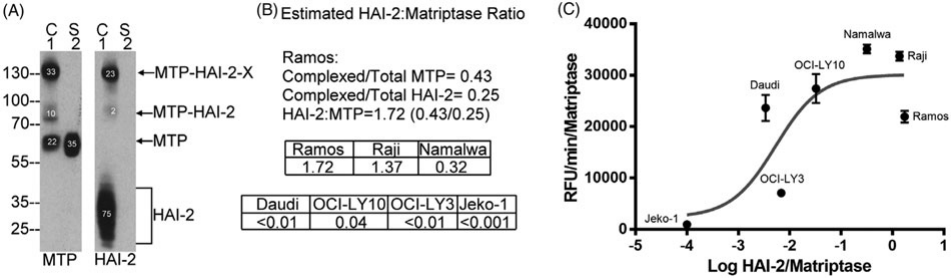
High HAI-2 expression does not suppress the levels of shed active matriptase in a proportional manner. The HAI-2:matriptase molar ratio was estimated in Ramos cells (A and B). Matriptase species and HAI-2 species in the cell lysate and conditioned buffer in Ramos cells were determined by western blot as described in Figure 4(A). The expression levels of matriptase species (A: left panel MTP) and HAI-2 species (A: right panel HAI-2) in the cell lysate (A: lanes 1, C) and the shed fraction (A: lanes 2, S) were determined by the relative signal density of each protein band, as indicated. The HAI-2:matriptase molar ratio in Ramos cells was calculated to be 1.72 (0.43/0.25 = 1.72), based on the ratio of matriptase-HAI-2 complexes relative to total matriptase (0.43) or to total HAI-2 (0.25), as indicated (B). The HAI-2:matriptase ratio in the remaining six neoplastic cells was then estimated based on the protein expression levels of matriptase and HAI-2, as shown in Figure 3(A,B), respectively. The rates of shed matriptase proteolytic activity normalised for matriptase expression level in the seven neoplastic cell lines were then plotted against the logarithm of their respective HAI-2:matriptase ratio (C).

In order to characterise the relationship between the level of HAI-2 protein expression and the level of shed matriptase activity in a more quantitative fashion, HAI-2:matriptase molar ratios were determined by taking advantage of the formation of stable activated matriptase complex with HAI-2 and the detection of these complexes with both the matriptase mAb and the HAI-2 mAb (Figure 6(A)). The HAI-2:matriptase molar ratio can be easily estimated by comparing the ratio of the signal intensity of matriptase-HAI-2 complexes relative to the signal intensity of total matriptase (0.33 + 0.1 = 0.43 of total matriptase, Figure 6(A) MTP) with the ratio of the signal intensity for matriptase-HAI-2 complexes relative to the signal intensity of total HAI-2 (0.23 + 0.02 = 0.25 of total HAI-2, Figure 6(A), HAI-2). The HAI-2:matriptase ratio was determined to be 1.72 (0.43/0.25 = 1.72) for Ramos cells (Figure 6(B)). Ramos cells were chosen for this analysis since they express similar levels of HAI-2 and matriptase, which made it easier to maintain the signal intensity in a relatively linear range, which is important for the accuracy of the estimation of the molar ratio. The very low level of HAI-2 in some of these cell lines and the great disparity in the levels of expression of HAI-2 and matriptase make it very hard to directly estimate the HAI-2:matriptase ratio in those lines. Having established the ratio in Ramos cells (1.72), we can, however, use the ratio as reference in conjunction with measurements of the relative matriptase protein expression levels (Figure 3(A)) and the HAI-2 protein expression levels (Figure 3(B)) to estimate HAI-2:matriptase ratio in the other six lines and shown in Figure 6(B). These estimated ratios indicate that Ramos and Raji cells express HAI-2 at a slightly higher level than matriptase; the HAI-2:matriptase ratio drops to 0.32 in Namalwa cells; HAI-2 is expressed at around or less than 1% of the level of matriptase in OCI-LY3, OCI-LY10 and Daudi cells; and Jeko-1 cells essentially express no HAI-2. The accuracy of this estimated ratio, at least for Namalwa in which matriptase is estimated to be expressed at three times the level of HAI-2, is supported by the depletion of HAI-2 monomer by active matriptase post-induction of matriptase zymogen activation (Figure 4(B), HAI-2, lane 3).

The HAI-2:matriptase ratios were then plotted on a log scale against the rates of matriptase proteolytic activity shed by the seven neoplastic B-cell lines (Figure 6(C)). The profile suggests a complicated relationship between shed active matriptase and HAI-2 expression. Parts of this complexity likely result from the inability, or significantly compromised ability of Jeko-1 and OCI-LY 3 cells to undergo matriptase zymogen activation, because HAI-2 is either not expressed or expressed primarily as an “immature” form. Furthermore, the conventional Yin and Yang relationship between protein protease inhibitors and their target proteases does not appear to hold for the remaining five lines. Daudi and OCI-LY10 cells express a very low level of HAI-2, which does not result in much higher levels of shed matriptase proteolytic activity. While higher HAI-2 expression does appear to result in less shed matriptase proteolytic activity when comparing Ramos to Namalwa and Raji cells, the reduction in shed matriptase proteolytic activity is not really in proportion to the HAI-2:matriptase ratio. For example, the rate of shed matriptase proteolytic activity from Ramos cells is around 62.5% of that from Namalwa cells (7443 versus 11,903 RFU/min/matriptase). This roughly 40% reduction is out of proportion to the 5.4 (1.72/0.32) fold higher HAI-2:matriptase ratio in Ramos cells compared to Namalwa cells. Taken together, these analyses suggest that matriptase zymogen activation may be somewhat compromised in neoplastic B-cells that do not express or express very low levels of HAI-2, or primarily express immature HAI-2. Likewise, higher HAI-2 expression may not lead to a proportional suppression of matriptase enzymatic activity shed from the cells.

## Discussion

The ectopic expression in some haematological cancer, particularly in B-cell neoplasms, but not in normal lymphocytes indicates that matriptase might have the potential to contribute to the development and progression of the disease. Much of the pathophysiological role of matriptase relies on its enzymatic activity. In the current study, the major regulatory mechanisms involving the control of matriptase proteolytic activity were assessed. Expression analysis at the mRNA level reveals that matriptase could be differentially regulated in neoplastic B-cells compared to carcinoma cells, in which both HAI-1 and HAI-2 were almost ubiquitously co-expressed at high levels. In neoplastic B-cells, matriptase is co-expressed with both HAI-1 and HAI-2 together, or HAI-2 alone, or HAI-1 alone. In general, the expression levels of HAIs are lower in neoplastic B-cells than in carcinoma cells. An imbalance favouring matriptase proteolysis would, therefore, more likely to happen to neoplastic B-cells. A previous study has shown that imbalanced matriptase proteolysis converts the physiological serine protease into an oncogenic one, resulting in the development of squamous cell carcinoma in the skin of all matriptase transgenic mice^8^. Besides the differential expression of HAIs, matriptase enzymatic activity appears to be greatly affected by the differential abilities of these cells to undergo zymogen activation. An unexpected finding was that HAI-2 appears to not be an effective inhibitor to control the levels of extracellular active matriptase in spite of the fact that HAI-2 is a very potent matriptase protease inhibitor in solution.

Matriptase is synthesised as an inactive zymogen. Whether the cells possess the ability to undergo zymogen activation represents one of the most important factors which determine the levels of enzymatically active matriptase. The inability of Jeko-1 to undergo zymogen activation highlights the mechanism to be a prerequisite in the functional regulation of the type 2 transmembrane serine protease. The very high level of matriptase zymogen protein in conjunction with the resultant low level of shed active matriptase in OCI-LY3 provides another example for the importance of the ability to undergo zymogen activation in matriptase regulation. The importance of zymogen activation over the level of matriptase protein expression can be also manifested by the similar levels of active matriptase shed (Figure 5(C)) versus the large difference in the level of matriptase zymogen protein expressed (Figure 3(A)) prior to the induction of zymogen activation between OCI-LY 3 and Raji cells. Matriptase zymogen activation is carried out through an unusual autoactivation mechanism which depends on the weak matriptase zymogen intrinsic activity^11^. In addition to induction by exposure to a mildly acidic pH environment, matriptase zymogen activation can be positively or negatively regulated by redox environment, certain chemicals, steroid hormones, lysophospholipids, and proteins factors in cell-type selective or nonselective ways^14^^,^^16^^,^^29^^,^^38^^,^^39^. The reason for the lack of, or the compromised ability of Jeko-1 and OCY-LY3 to activate matriptase in response to the mildly acidic buffer remains unclear. Previous studies using exogenous expression models indicate that both HAI-1 and HAI-2 have functions beyond their role as matriptase enzymatic inhibitors. Both HAIs appear to affect matriptase expression, intracellular trafficking, and zymogen activation. Given the negligible expression of the HAIs or the expression of HAI-2 primarily in the lightly N-glycosylated form in these two neoplastic B-cells, it is of interest to investigate in the future if the status of HAI expression and N-glycosylation affect their ability to activate matriptase. It is worth noting that OCI-LY 3 cells have an in-frame deletion of *ALG3*, which encodes alpha-1,3- mannosyltransferase (UCSC Xena, https://xena.ucsc.edu/). Defects in *ALG3* have been reported to cause abnormal N-glycosylation^40^. The neoplastic B-cells could be used as a model to investigate the role of HAI-2 and its N-glycosylation status in matriptase zymogen activation.

Among those cells with “normal” ability to activate matriptase, the level of active matriptase shed into the extracellular milieu was initially thought to depend on a balancing act between the levels of matriptase protein expressed and the ratio of matriptase relative to the endogenous protease inhibitor, HAI-2 here. While very different levels of active matriptase were detected from the same number of cells among OCI-LY 10, Daudi, Namalwa, Raji, and Ramos cells (Figure 5(C)), the difference in the levels of shed active matriptase was correlated more with matriptase protein levels than the HAI-2:matriptase ratio among these 5 cell lines. These observations are in one way consistent with the conventional belief that more matriptase expression will have more active matriptase, but in another way are not consistent with the functional relationship between protease and protease inhibitor: more protease inhibitor less protease activity. While this could result from some unknown variation among the five different neoplastic B-cells, our preliminary observation indicates a potentially paradoxical role for HAI-2 in matriptase regulation. In order to avoid the cell line variation, the ineffectiveness of HAI-2 in the control of matriptase enzymatic activity could be tested in the future by expressing different levels of HAI-2 in a cell line with extremely low HAI-2 expression, such as Daudi.

In conclusion, matriptase enzymatic activity could be regulated by several different mechanisms, including matriptase expression levels, the ability to undergo zymogen activation, and the ratio in relation to HAIs. These three major mechanisms could vary significantly among different neoplastic B-cells with a trend by which matriptase proteolysis could be enhanced by the lower HAI expression and the ineffectiveness of HAI-2 in the control of extracellular matriptase activity.
